# Charcot’s Method of Treating Progressive Locomotor Ataxia by Suspension

**Published:** 1889-04

**Authors:** Edward B. Angell

**Affiliations:** Rochester, N. Y.


### *CHARCOT'S METHOD OF TREATING PROGRESSIVE LOCOMOTOR ATAXIA BY SUSPENSION.*

Abstract.—Translated by EDWARD B. ANGELL, M. D., Rochester, N. Y.

*Le Progrès Médical*, of February 23d, contains an article by Gilles de la Tourette, descriptive of the technique employed by Charcot in the treatment of progressive locomotor ataxia by suspension, recently promulgated by Motchoukowsky, of Odessa. As this plan promises to be of considerable value in relieving this obdurate malady, a résumé of the *modus operandi* will be of interest.

The means employed is the well-known Sayre's suspension apparatus, which does not demand special notice.

The various parts of the apparatus must be carefully adjusted, in order to secure satisfactory results. Either extremity of the yoke, or suspending bar, should have two or three notches, in order to allow for the head-sling sufficient adjustability to different-sized heads. It is important that traction during elevation does not wear solely upon the head and neck, as suspension could not be tolerated. The body must have a further point of support, which, however, must not interfere with the stretching of the vertebral column. Hence, slings for the axilla are provided and made adjustable to the varying height and weight of the patients. If the axillary piece is too short, compression of the brachial plexus with its attendant evils is produced, necessitating an interruption of the séance. If the sling is too long, too great a strain comes upon the neck, and the suspension becomes unendurable. Indeed, careful experiment must be made in order to determine the satisfactory fit of the apparatus, or harm will follow rather than good. Ordinarily as used, the yoke is hung from a portable tripod. But for this purpose it is preferable to swing it from a stationary hoop in the ceiling, since cases of ataxia are so unstable of station that they are liable, through fear of losing their equilibrium, to grasp the tripod legs convulsively and overturn it. Then with everything in readiness, the patient is slowly and progressively drawn upward, so as to accustom the muscles of the neck to the traction. The patient is cautioned against making movements, especially involuntarily ones, when he feels himself leaving the ground, in order to avoid lateral displacement or torsion.

When the patient is swung up so that the ends of the feet, turned downward, do not meet the ground, he is lightly held to prevent twisting, while the time is carefully noted, and the length of each séance minutely regulated.

He is ordered from time to time to raise his arms slowly and vertically, in order to make this traction still more efficient.

Generally, the longest séance ought not to exceed three or four minutes, three minutes being perhaps the average.

The duration of the first trial should last only half a minute, and be increased gradually to the limit mentioned by the sixth or eighth séance.

Further, it is necessary to consider individual peculiarities, especially that of weight. While, for example, two minutes of suspension may be tolerated by a patient weighing 150 to 200 pounds, it is different with those weighing 200 or more.

In these cases of excessive weight, the traction which comes upon the neck is very hard and painful perhaps throughout the day following the suspension. This ought not to be the case if the operation is carefully made.

Among some patients the desire to get well is so strong that they think they are obliged to suffer, without complaint, that which is too painful to secure good results.

The operation ought to cause neither pain nor fatigue, under penalty of being useless. Hence, while patients of light weight may readily bear three and a half minutes of suspension, heavier ones should not go beyond three minutes. Yet with them the traction, as may be readily understood, is very efficient.

The sêances ought to be every other day, experience having shown that daily sêances were more painful than useful. The time of day matters little, but regularity is indispensable.

At the expiration of the time, the physician relaxes the cord little by little, and the patient reaches the ground slowly without concussion. He is then relieved of his harness, and immediately seated for a short time to allow some rest.

The patient ought to be divested of his upper garments, in order to admit free play of the arms; the neck should be bare, or at least not surrounded by a tight collar.

The results so far obtained have been very remarkable in progressive locomotor ataxia, in a case of Friedrich's disease, with a jacket to correct the deviation of the vertebral column, and in two cases of neurasthenia, with marked sexual impotence, they have been satisfactory.

On the other hand, suspension has rather aggravated the symptoms of spasmodic paraplegia in a case of insular sclerosis. Two patients affected with paralysis agitans, seem also to have received some benefit. However, in regard to progressive locomotor ataxia, particularly is it difficult to formulate very exactly the indications for treatment, the number of cases at present being too limited to yield a definite opinion. Probably it is certain it should not be applied in all instances; the recent cases seeming to receive less benefit, though some of the symptoms are particularly influenced in a happy manner. This selection itself is a proof of the therapeutic value of a method which makes no claims to being a panacea. What can be said is this: Charcot has obtained among the patients who have been coming to the Saltpêtrière for many years, very encouraging improvement, especially where the malady has seemed to defy therapeutic treatment. Likewise, is it difficult to pronounce upon the duration of treatment, since the first ataxies who were submitted to suspension at the Saltpêtrière four months ago, are still under treatment, and continue to gain in a progressive manner. In all cases, as Charcot has said, the trial may be made confidently, as it has always appeared, when satisfactorily applied, wholly uninjurious.

